# The Influence of Welfare Training on Bird Welfare and Carcass Quality in Two Commercial Poultry Primary Processing Plants

**DOI:** 10.3390/ani9080584

**Published:** 2019-08-20

**Authors:** Ellie Wigham, Andrew Grist, Siobhan Mullan, Stephen Wotton, Andrew Butterworth

**Affiliations:** Bristol Veterinary School, University of Bristol, Langford, Bristol BS40 5DU, UK

**Keywords:** animal welfare, broilers, slaughter, meat quality, welfare training, welfare assessment

## Abstract

**Simple Summary:**

Ensuring acceptable welfare of broilers at the slaughterhouse is paramount in meeting legislative and retailer specifications, and in producing high quality meat. Animal welfare training for staff working in red meat slaughterhouses has been shown to improve animal welfare measures and product quality, however there is little evidence of the effects of welfare training in poultry processing plants. The aim of this study was to investigate the impact of introducing a welfare training program for staff on bird welfare and carcass quality in a commercial Costa Rican and British poultry slaughter plant. The results we obtained show that staff welfare training has a positive effect on several bird welfare outcomes. Some carcass quality measures also improved post training, although this result was not consistent. These data could help the development and targeting of future welfare training courses and encourage the uptake of welfare training in the poultry slaughter industry.

**Abstract:**

The number of broilers slaughtered globally is increasing. Ensuring acceptable welfare conditions for birds at the time of slaughter is paramount in meeting legislative and retailer specifications, and in producing high quality meat. There is knowledge that welfare training programs for members of the farming and red meat slaughter industry can improve animal welfare measures and product quality, however there is little evidence of the effects of welfare training in poultry processing plants. In our study, a comprehensive welfare training program was introduced to a Costa Rican and a British commercial broiler primary processing plant, both of which slaughter birds by way of neck cut post electrical water bath stunning. The effects of this program on some welfare and product quality measures were investigated, both immediately and six months post training. The welfare measures that showed significant improvements post training included; flapping at shackling, pre-stun shocks, stun parameters and effective neck cut. Product quality measures including broken wings and red pygostyles also improved, however the positive effect of training was not seen in all quality measures. Welfare training does have the potential to improve broiler welfare and product quality at slaughter, and these data could help the development and targeting of future welfare training courses and encourage the uptake of welfare training in the poultry slaughter industry.

## 1. Introduction

The worldwide consumption and production of poultry meat is increasing; It is estimated that in 2018 global output reached 121.6 million tonnes, an increase of 1.4% on 2017 [1]. In order to meet demands, tens of billions of broilers are slaughtered every year, and the welfare of these animals is a growing concern for the public [2], retailers [3] and slaughter business operators [4]. Those involved with the routine killing of animals for food production have both an ethical obligation and the practical opportunity to minimise any associated suffering with each animal that is killed [5].

From arrival to death, bird welfare can be affected by each process within a commercial slaughter facility, and while producing high quality poultry meat on a commercial level requires a multi-factorial approach [6,7] there are well documented associations between bird welfare during these pre-slaughter processes and carcass quality [6,8]. For example, violent wing flapping in shackled birds may be viewed as an index of discomfort [9]. At the point of shackling, wing flapping is associated with rough handling and compression of the birds’ hock due to tight fitting shackles [9,10]. Violent wing flapping can also occur as a result of pre-stun shocks when birds enter the water bath stunner, [11] (a painful electric shock occurring when any part of the bird makes contact with electrically-live water bath prior to head entry) [9]. This flapping behaviour is associated with quality defects such as red wing tips [12], broken wings and wing haemorrhages [13,14]. These conditions can lead to product downgrading, and thus can be economically significant for slaughter business operators [15].

Similarly, poor neck cutting has both welfare and product quality consequences. Inadequate neck cutting can result in birds regaining consciousness during bleeding. Ideally the cut should severe all major blood vessels in the neck of the bird [16] particularly the two carotid arteries which supply oxygenated blood to the brain [17,18]. Poor cutting may lead to poor bleed out, resulting in residual blood in carcass pygostyles, shoulders [19] and wings [20] which appear as haemorrhages post plucking. It should be noted that rough handling of birds by slaughterhouse operators during any pre-slaughter activity has links to product quality defects, such as shoulder and wing haemorrhages [21], broken wings [22] and bruised thighs [23], all of which are a cause of pain and suffering in live birds.

There is evidence that animal welfare training has the capacity to improve animal welfare on farm [24] and in red meat abattoirs [25,26,27]. It has been suggested that such training may also improve the welfare of broilers at slaughter [28] thus having the potential for improved carcass quality, however there is a lack of published evidence of such effects. This study aimed to gain an understanding of the influence that the introduction of a welfare training course for abattoir staff may have on bird welfare and product quality in commercial poultry slaughter facilities, an understanding of which may benefit the development and targeting of future welfare training courses and encourage the uptake of welfare training in the poultry slaughter industry. To the authors’ knowledge, this is the first study to outline the effects of staff training in such an environment using some animal-based measures.

## 2. Materials and Methods

The study took place between January 2018 and January 2019. A comprehensive welfare training course was introduced in two commercial broiler primary processing plants. Welfare and product quality assessments were performed prior to training (pre-T), immediately post training (post-T) and six months post training (6mpost-T). 

### 2.1. Primary Processing Plants

Two primary processing plants were involved in the study, one situated in Costa Rica (Processing Plant P1) and one in the UK (Processing Plant P2). Both facilities used electrical water-bath stunning. Processing Plant P1 operated two shifts whilst Processing Plant P2 operated one shift. Their individual characteristics can be found in Table 1. 

### 2.2. Training

Both primary processing plants received the same training program (with the exception of legislative information which was country specific). The training was based on the Poultry Welfare Officer (PWO) Training Course run by the University of Bristol which have been designed to deliver continued professional development to the meat industry and provides individuals with the technical competence to achieve Animal Welfare Officer (AWO) status in poultry slaughter plants.

The courses were delivered by an experienced trainer from AWO Training Langford (University of Bristol).

Plant management received a two-day comprehensive classroom-based training program which covered the following topics; legislation, catching, transportation, lairaging, emergency slaughter, hang-on, effective stunning, influence of welfare on product quality and poultry slaughter. The training sessions consisted of lectures interspersed with group discussions and quizzes. A total of 26 management personnel attended the training from Processing Plant P1 and 11 management personnel from Processing Plant P2. (The greater number of attendees from P1 is to account for the two-shift system run by this plant.)

Operatives, including those responsible for lairaging, shackling and neck cutting of live birds, received a 20-minute group training session delivered by AWO Training Langford (University of Bristol) consisting of an interactive, multi-media-based lecture outlining “better practice” in bird handling, shackling technique and neck cutting. All operatives employed by the plants at the time of the study received training (including staff working on both shifts in Plant P1). Training for operatives was conducted prior to the start of their shift.

### 2.3. Timeline of Assessments

**Figure 1:**
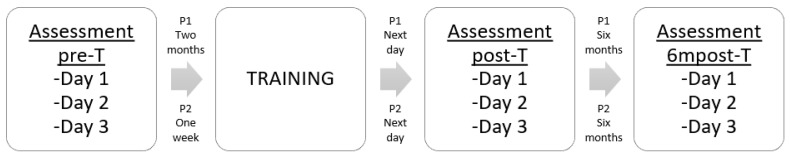
Outline of the study.

Each primary processing plant was assessed on three occasions; once prior to training (two months prior to training at plant P1 and one week prior to training at plant P2); once immediately post training (assessment commencing the day after training was completed) and once exactly six months after training. Each assessment visit lasted three days and both the welfare assessment and the product quality assessment were repeated on each day (Figure 1). Due to the potential to disrupt production, the stun parameters and neck cut were only assessed on day one of each visit. The assessments were carried out on the same days of the week, and at the same time of day during each visit. Plant management were aware that the assessments were taking place. Although operatives were not specifically told that welfare assessments were being undertaken, they were aware that they were being observed.

### 2.4. Welfare Assessment

Aspects of bird welfare during five elements of pre-slaughter and slaughter operations were investigated. Due to the speed of the slaughter line, it was not possible to observe the same birds at each stage of the assessment.

#### 2.4.1. Lairaging

Birds in the lairage of processing plant P1 were contained in plastic transport crates while those in processing plant P2 were contained in an Anglia Autoflow Easyload Drawer System. Twenty crates/drawers were scored for the presence of panting birds. Due to the difficulty in observing every individual bird within drawers or crates without disturbing the animals, a drawer or crate was scored “positive” (+ve) for heat stress if one or more birds which it contained, was observed panting.

In both processing plants the crates/drawers were stored in stacks, seven crates/drawers were observed at the top of stacks, seven at the bottom and six in the middle. Stacks were observed in different areas of the lairage, however capacity to do this varied depending on the number of stacks present at the time of sampling.

The daily environmental temperature and relative humidity of the lairage were measured using a Kestrel 4000 Pocket Weather Tracker prior to the start of observations. At plant P1 observations commenced at 7:30 p.m. At plant P2 observations commenced at 9:30 a.m.

#### 2.4.2. Hang on

Each operator hanging birds in the shackling area was observed handling 100 birds. The number of birds vigorously flapping (prolonged, >2 s, bout of rapid wing flapping) immediately after the hang-on operator completely removed both hands from the bird was recorded at each operator position. The operator shackling birds closest to the water bath stunner was deemed as working at position 1 with each successive operator occupying subsequent positions. Prior to entering the water bath, birds were shackled for a time ranging 9 to 16 seconds in plant P1 and 20 to 27 seconds in plant P2.

#### 2.4.3. Entering Stun Bath

The entry of 500 birds into the electrical water bath stunner was assessed for pre-stun shocks (PSS). The birds were scored based on the protocol described by Rao, Knowles and Wotton [13].

- Score 0 = an uninterrupted entry into the water bath where only a single contraction of the skeletal muscles occurred.
- Score 1 = more than one separate contraction in response to electrical stimulation.
- Score 2 = the bird lifts its head and flies the first stage of the water bath.

#### 2.4.4. Stun Parameters

A factory calibrated poultry stun monitor (PSM—AGL Consultancy Ltd.) was used to measure the true root mean square (RMS) current being applied to a known resistor (1000 Ω) in the water bath stunner. The PSM was hung on the shackles at the shackling point and passed through the bath at the operating line speed. This was repeated six times during normal production i.e., with birds present in the water-bath. 

The frequency setting of the water-bath was not measured, however, the programmed setting on the stunner control panel was noted. 

#### 2.4.5. Neck Cut

Fifty birds were selected at random during the bleeding process and removed from the line. Blunt dissection of the neck was carried out to allow a visual examination of the carotid arteries on each side of the neck. A record was made of whether these vessels were intact or severed. 

### 2.5. Product Quality Assessment

Measures of product quality included in the assessment were selected based on their association with bird welfare at slaughter.

The product quality assessment was undertaken immediately after completion of the welfare assessment. All external scoring (carcass quality assessment) was carried out by the same individual using a subjective comparison against photographic standards [15]. Two-hundred carcasses were assessed for each carcass quality characteristic. As the inspection took place on the moving production line, a different set of carcasses were assessed for each characteristic. 

Following automated scalding and plucking, and whilst still on the primary processing shackle line, carcasses were scored for external quality. The presence of broken wings with an associated haemorrhage and leg bruising was noted (0 or 1). Due to the potential of wings to be broken by the plucking process, which is not a concern for welfare, wings were only scored as broken if the damage was associated with a haemorrhage as this indicates that the damaged occurred pre-slaughter. Red pygostyles were scored on a scale from 0 (no bruising) to 2 (severe bruising) and red wing tips, shoulder haemorrhage and wing haemorrhage, were all scored on a scale from 0 (no damage) to 3 (severe damage). 

For each quality measure, with the exception of leg bruising, the carcass was given an overall score. If there was a discrepancy between the wing scores (right and left) of an individual carcass, the overall score would be the higher of the two e.g., if one wing of an individual carcass scored 0 for the presence of red wing tips, while the other wing scored 2, the carcass score would be 2. Carcasses were scored positive for broken wings if either one or both wings were broken. Scores of 2 and 3 represent levels of damage that result in carcass downgrading leading to economic losses for the processing plant [29]. 

Each leg received an individual score (rather than an overall carcass score). This is to account for the potential impact that one-leg catching techniques (whilst harvesting the birds from the farm) may have on the incidence of bruising. 

### 2.6. Statistical Analysis

To assess the significance of the training events on both animal welfare and product quality metrics, statistical analysis of the data collected and collated during the visits was performed using SPSS vs. 24.0. Graphs were plotted using Microsoft Excel. Analysis was carried out separately for each primary processing plant. Results were deemed significant at *p* ≤ 0.05 level.

#### 2.6.1. Welfare Assessment Analysis

The difference between visits on the number of crates/drawers containing panting birds was tested using the Kruskal–Wallis test. The relationship between environmental temperature, relative humidity and the number of crates/drawers containing panting birds was tested using Spearman’s rank-order correlation. Whether training had an influence on operator shackling was investigated using a univariate general linear model (GLM). The dependent variable in the GLM was the number of birds observed vigorously flapping at each operator position against the fixed factors of the visit. Data from the three observation days of each visit were combined to give a total number of flapping birds at each operator position during each visit. 

The difference between the visits in the proportion of birds flapping at three operator positions (Position 1, Position 3, Position 6) was calculated and the significance of this difference was investigated using an exact Chi square test. 

Kendall’s tau-b statistic was used to test for an association between number of birds receiving each pre-stun shock score during different visits (tested in pairs: pre-T–post T; pre-T–6mpost-T; post-T–6mpost-T). The daily counts were combined to give a total for each visit. The proportion of birds receiving shocks (categories PSS1 and PSS2 combined) and proportion of birds receiving severe shocks (PSS2) was calculated for each visit. The significance in the difference in proportion of birds receiving each type of shock between the visits was investigated using an exact Chi square test. To investigate differences in PSM readings between visits, a one-way ANOVA with Tukey post hoc analysis was performed. Due to small sample sizes (n = 3), the effects of welfare training on effective neck cutting was assessed by a visual inspection of plots.

#### 2.6.2. Product Quality Assessment Analysis

For each product quality measurement, daily score counts were combined to give a total score count for each assessment visit. To assess the difference between visits, a cross-tabulation of the number of birds in each quality outcome category broken down by visit was produced for each quality measure. Each table was tested for the association between the counts in each quality category and visit, by means of a Chi-square test, for binary outcomes measures, or by using Kendall’s tau-b statistic for those with ordered three or four category outcomes. Visits were tested in pairs (pre-T–post T; pre-T–6mpost-T; post-T–6mpost-T). Exact statistics were calculated in all cases.

For quality measurements which were made on a scale of 0 to 2 or 0 to 3, levels 0 and 1 are considered to have no economic consequence but levels 2 and 3 will result in downgrading [29]. For these scales the levels 0 and 1 and 2 and 3 (where applicable) were collapsed to give a binary variable signifying no economic consequence (0) or damage of economic consequence (1). In this way all outcomes measures become binary variables and therefore subjected to a secondary analysis using a Chi-squared test as described above.

## 3. Results

### 3.1. Welfare Assessment

#### 3.1.1. Lairaging

**Figure 2:**
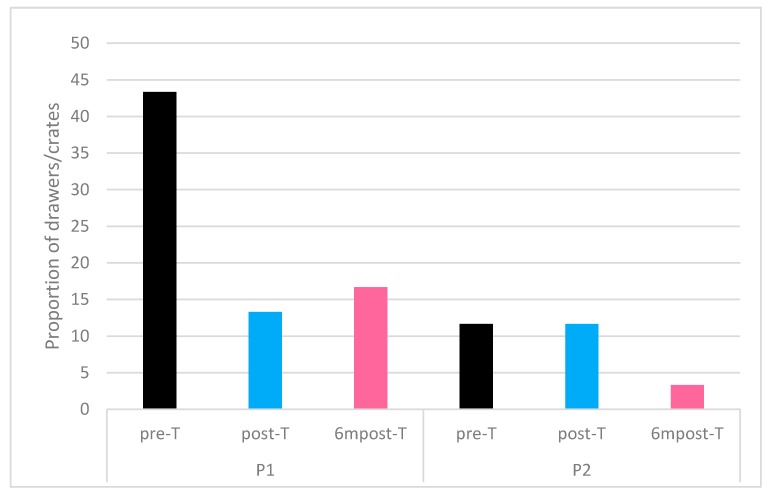
Proportion of crates/drawers containing panting birds n = 60.

The proportion of crates/drawers containing panting birds out of the 60 observed each visit is shown in Figure 2. Temperature and relative humidity measurements are presented in Table 2.

A Kruskal–Wallis test showed no significant difference in the proportion of crates/drawers containing panting birds between the visits in both processing plants P1 (χ^2^(2) = 0.807, *p* = 0.668) and P2 (χ^2^(2) = 1.272, *p* = 0.529).

In processing plant P1 the proportion of crates containing panting birds was not significantly correlated with lairage temperature (r_s_(7) = 0.363, *p* = 0.337) or relative humidity (r_s_(7) = −0.126, *p* = 0.747). Sprinkler fans were in use in the lairage of processing plant P1, it was observed that the sprinkler was not in use during post-T.

Processing plant P2 also had no significant correlation between lairage temperature (r_s_(7) = 0.324, *p* = 0.396) or relative humidity (r_s_(7) = 0.184, *p* = 0.636) with the proportion of drawers containing panting birds. In the lairage of processing plant P2, it was observed that trucks were often being washed in close vicinity to the stacks of drawers.

#### 3.1.2. Hang on

Processing plant P1 had six operators shackling birds, processing plant P2 had seven operators shackling birds.

Operator position was a significant predictor of the number of birds vigorously flapping immediately after hang-on, in both processing plant P1 (F (_1,48_) = 91.244, *p* < 0.0005) and P2 (F (_1,57_) = 57.18, *p* < 0.0005).

Visit was not a significant predictor of the number of birds vigorously flapping immediately after hang-on in processing plant P1 (F (_1,48_) = 46.445, *p* = 0.634) or P2 (F (_1,57_) = 1.507, *p* = 0.230).

There was a significant position × visit interaction effect in processing plant P1 (F (_1,48_) = 10.067, *p* < 0.0001) but not in P2 (F (_1,57_) = 0.374, *p* = 0.69).

To further explore the interaction effect in processing plant P1 the difference in proportion of birds flapping at each visit was investigated. To account for the effect of other factors which may influence flapping (e.g., number of birds already on the shackle line at the point of hanging) it was decided to investigate the impact of visit at three operator positions; at the position closest to the water bath (position 1); in the middle of the hang-on area (position 3) and at the position furthest from the water bath (position 6).

**Figure 3:**
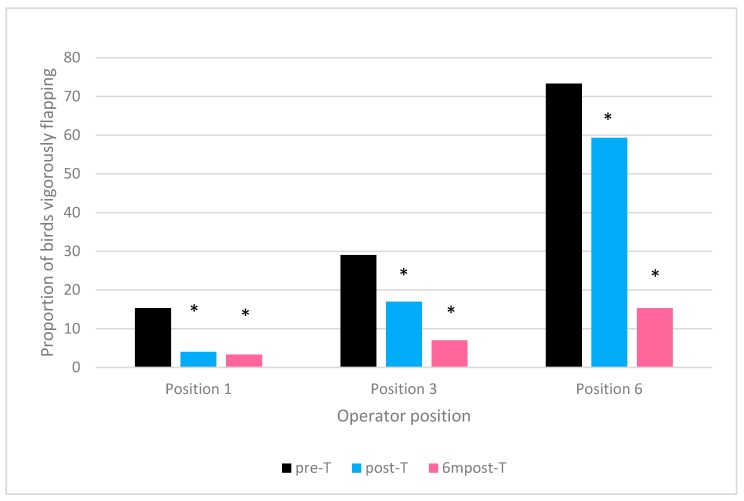
Proportion of birds vigorously flapping at hang on at different operator positions for each visit (Processing Plant P1). * represents a statistically significant difference in proportion from pre-training visit (*p* ≤ 0.05) calculated using the exact Chi-squared test.

At each of the investigated positions, greater proportions of birds flapped after hang-on prior to training compared to post training. Proportions were lower six months post training compared to immediately after training (Figure 3). 

A statistically significant difference in proportion of birds performing vigorous flapping was found in all post-training visits compared to pre-training values. The greatest differences were found at position 6 between pre-T and 6mpost-T in which flapping decreased by 58%. 

#### 3.1.3. Entering Stun Bath

Kendall’s tau-b statistic and *p* values for the associations between PSS scores and pairs of visits is given in Table 3. These indicate that there was a difference between every visit pair in the proportion of PSS scores in processing plant P1. Processing plant P2 showed a difference between pre-T and 6mpost-T and between post-T and 6mpost-T but not between pre-T and post-T. 

**Figure 4:**
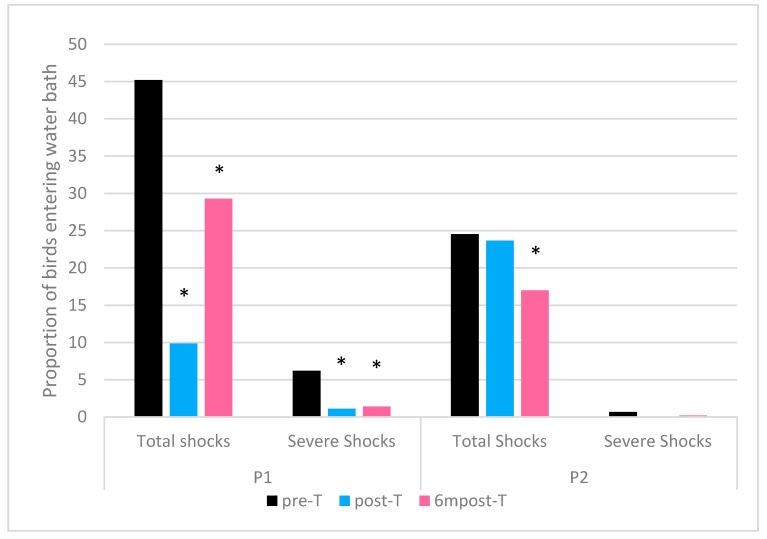
Proportion of birds receiving a pre-stun shock (score PSS1 and PSS2 combined) and severe pre-stun shocks (score PSS2) in both plants across each visit. * represents a statistically significant difference in proportion from pre-training visit (*p* ≤ 0.05) calculated using the exact Chi-squared test.

In processing plant P1 the proportion of birds receiving a pre-stun shock significantly decreased by 35.3% between pre-T and post-T, and by 15.9% between pre-T and 6mpost-T, however this proportion increased by 19.4% between post-T and 6mpost-T. The proportion of birds receiving severe shocks decreased by 5.1% between pre-T and post-T and by 4.8% between post-T and 6mpost-T (Figure 4). 

There were less marked differences in processing plant P2 with no significant change between the proportions of birds receiving PSS in pre-T and post-T. There was a significant decrease of 7.5% between pre-T and 6mpost-T and of 6.7% between post-T and 6mpost-T. Severe shocks were less affected, with a decrease of 0.6% between pre-T and post-T and no significant difference between pre-T and 6mpost-T (Figure 4).

#### 3.1.4. Stun Parameters

During all visits the frequency of the electrical current in the water bath in processing plant P1 was set at 400 Hz and the frequency in the water bath in processing plant P2 was set at 1500 Hz.

**Figure 5:**
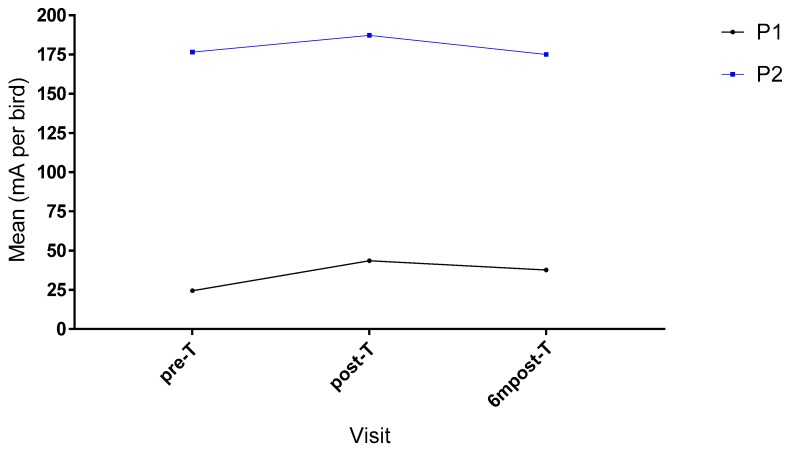
Mean PSM reading for each visit (n = 6 per visit) at processing plant P1 and P2. Error bars not included for clarity.

The mean PSM true RMS reading in mA per bird for each visit at both processing plants is shown in Figure 5.

The PSM current recorded in processing plant P1, showed a significant difference between visits, F (2,15) = 58.263 *p* < 0.0005 (one-way ANOVA). Tukey post hoc analysis was performed, showing that water bath current recorded by the PSM was significantly increased from pre-T to post-T by 19.0 (95% CI 13.1 to 24.91) mA per bird, *p* < 0.0005 and pre-T to 6mpost-T by 13.167 (95% CI 7.00 to 19.33) mA per bird, *p* = 0.001. However, the current had decreased significantly between post-T and 6mpost-T by 5.83 (95% CI 1.86 to 9.81) mA per bird, *p* = 0.011. 

The PSM current recorded in processing plant P2, showed a significant difference between groups F (2,15) = 15.697 *p* < 0.0005 (one-way ANOVA). Tukey post hoc analysis was performed, showing that water bath current recorded by the PSM was significantly increased from pre-T to post-T by 10.67 (95% CI 2.23 to 19.100) mA per bird, *p* = 0.018 but there was no significance between pre-T to 6mpost-T where it decreased by 1.5 (95% CI −9.93 to 6.932) mA per bird, *p* = 0.849. However, the current had decreased significantly between post-T and 6mpost-T by 12.167 (95% CI 8.68 to 51.65) mA per bird, *p* < 0.0005.

#### 3.1.5. Neck Cut

**Figure 6:**
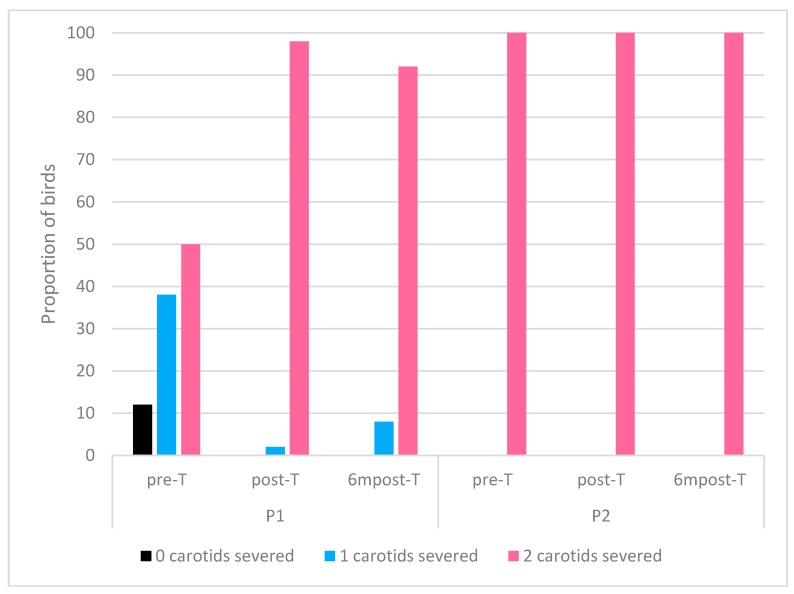
Proportion of birds per visit (n = 50) categorised by carotid arteries severed after cutting.

In processing plant P1, the proportion of birds with both carotids severed increased from pre-T to post-T and 6mpost-T. The proportion of birds with both carotids intact decreased to zero in post-T and 6mpost-T, while those with one severed carotid also decreased after pre-T (Figure 6). 

All birds inspected across all three visits in processing plant P2 had both carotids severed after neck cutting (Figure 6).

### 3.2. Product Quality Assessment

The results for the external product quality measurements given as the percentage of birds within each score category for each visit are given in Table 4 for processing plant P1 and Table 5 for processing plant P2.

**Figure 7:**
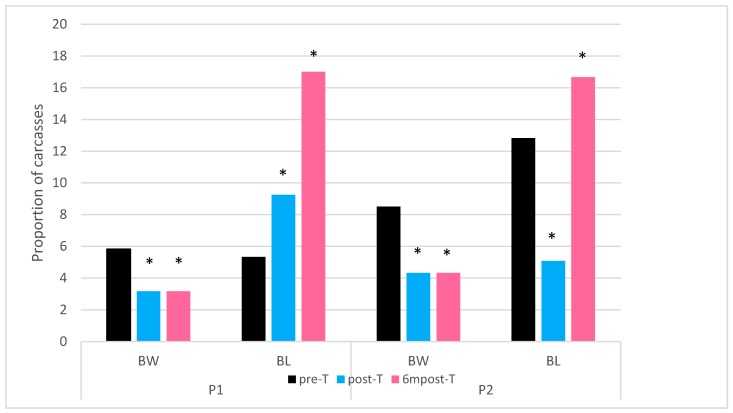
Proportion of carcasses with broken wings (BW) and bruised legs (BL). * represents a statistically significant difference in proportion from pre-training visit (*p* ≤ 0.05) calculated using the exact Chi-squared test.

In both processing plants, there was a significant decrease in the proportion of birds with broken wings observed in the post training visits compared to the pre-training visit. (Table 6 and Figure 7).

Conversely the number of bruised legs increased post training. In processing plant P1 there was a significant increase in bruised legs of 3.9% between pre-T and post-T and then a further increase of 7.8% between post-T and 6mpost-T. The proportion of birds with bruised legs in processing plant P2 decreased by 7.7% between pre-T and post-T, however, in 6mpost-T, the levels of bruised legs was 3.8% greater than those recorded in pre-T (Table 6 and Figure 7). 

**Figure 8:**
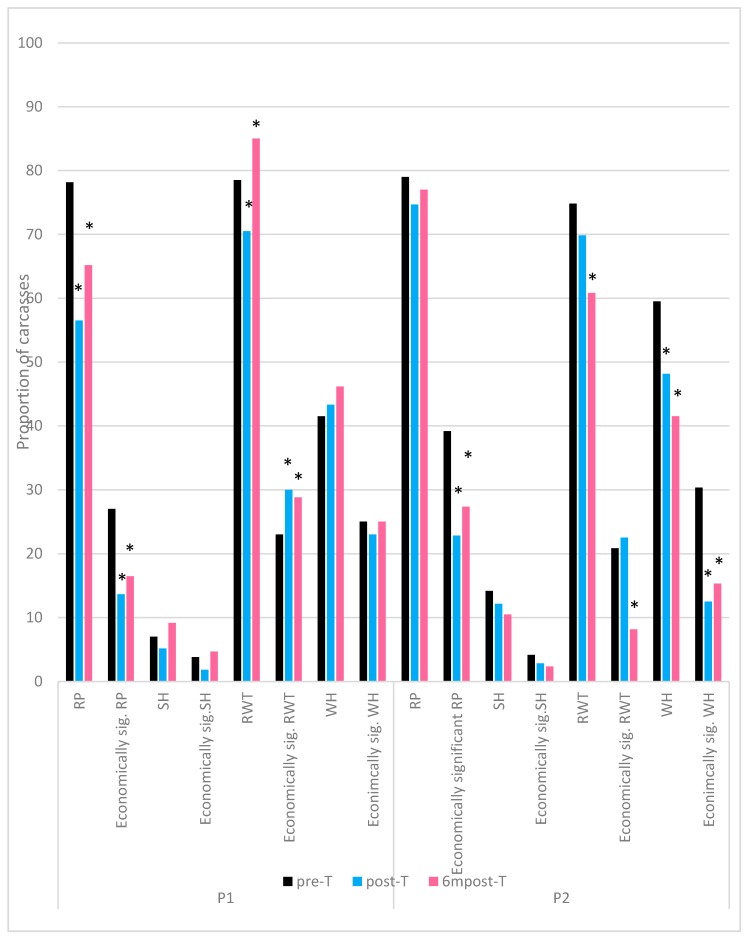
Proportion of carcasses with product quality defects (RP—red pygostyles; SH—shoulder haemorrhage; RWT—red wing tips; WH—wing haemorrhage) and economically significant quality defects (RP—quality assessment score 2; SH, RWT, WH—quality assessment score 2 and 3). * represents a statistically significant difference in proportion from pre-training visit (*p* ≤ 0.05) calculated using the exact Chi-squared test.

The tests of association between each visit, given in Table 6, indicate that there was a difference in the level of red pygostyles between all the visits, except for between post-T and 6mpost-T in processing plant P2. The proportion of carcasses with red pygostyles during each visit is given in Figure 8. In processing plant P1 levels of red pygostyles were lower in both post training visits compared to pre-training levels, however there were no significant changes in processing plant P2. Proportion of carcasses with severe red pygostyles (quality category 2) during each visit is also given in Figure 8. In both processing plants P1 and P2 levels of economically significant red pygostyles were significantly lower in both post training visits compared to pre-training levels.

The only significant change in the levels of shoulder haemorrhage was seen in processing plant P1 between post-T and 6mpost-T (Table 6) where there was an increase in proportion of 4% in overall bruising levels and an increase of 2.8% in economically significant bruising (Figure 8). 

There were no significant differences in the level of red wing tips between pre-T and post-T in processing plant P2, however Table 6 suggests there were differences across the remaining visits. 

The overall proportion of birds with red wing tips in processing plant P1 decreased by 9% between pre-T and post-T, however, the levels increased during 6mpost-T by 5.5% greater than during pre-training observations (Figure 8). Processing plant P2 displayed a significant decrease in red wing tips post training, with the decrease in 6mpost-T, 9% greater than in post-T. 

Economically significant red wing tips (quality measurement scores 2 and 3) had significantly increased in both post training visits compared to pre-training in processing plant P1. In processing plant P2 observed levels were significantly decreased in 6mpost-T compared to pre-T (12.7%) and post-T (14.3%) (Figure 8). 

There was no significant change in the proportion of overall wing haemorrhage, or economically significant wing haemorrhage between any of the visits in processing plant P1 (Table 6, Figure 8). In processing plant P2, the proportion of both overall wing haemorrhage and economically significant wing haemorrhage was significantly lower in the post training visits compared to the pre-training visit (Figure 8). 

## 4. Discussion

In this study, the effects of introducing a comprehensive welfare training program for plant management, alongside role specific training for operatives, was evaluated for impact on animal welfare and product quality in two commercial poultry primary processing plants. To the authors’ knowledge, this is the first study to outline the effects of staff training in such an environment, the impact of which has been assessed using animal-based measures. Understanding the influence of welfare training in slaughterhouses in both developed and developing countries may benefit future education courses by enabling the tailoring and targeting of welfare training programs, and by encouraging uptake within the slaughter industry. 

### 4.1. Welfare Assessment

Our study highlights that welfare training has the potential to improve animal-based welfare measures. Except for birds experiencing heat stress, the Costa Rican processing plant P1 improved in respect to all other welfare outcome measures. Enhancements were less marked in the British processing plant P2, however welfare improvements were seen in the numbers of birds experiencing PSS and in the current levels used for stunning. 

The number of birds experiencing heat stress in the lairage was the only welfare measure included in the study which did not significantly change between the visits to either processing plant. No correlation was found between the number of birds panting and environmental temperature or relative humidity. Quinn, et al. [30] concluded that due to the open nature of poultry lairages, and the activities which go on in them, many factors can influence the “quality” of the environment. Although the general atmospheric temperature and ventilation can be controlled, it is challenging to elicit changes at a bird level [30]. Large ventilation fans were present in the lairage of both processing plants and were in operation during all visits. It was observed that the fans in the lairage of processing plant P1 were installed with a sprinkler function which was in use during pre-T and 6mpost-T but was switched off during post-T. It is possible that the training influenced this change and may provide an explanation for the lower relative humidity recorded during post-T. Humidity readings in processing plant P2 were higher than expected given the environmental temperature. It was recorded that lorries delivering modules of broilers to the plant were often being washed in close vicinity to the stacks of drawers, thus contributing to the high humidity readings. 

A longer period in the lairage progressively increases bird body temperature. Warriss et al. [31] reported that birds killed four hours after arrival at a processing plant had a temperature 0.6 °C higher than those killed immediately on arrival, with an increase of 0.3 °C occurring during the first hour. Although, in this study, observations were taken at the same time during each day of observations, the period that the birds had been present in the lairage during the recordings was not known and would likely have differed between the visits. 

High stocking densities within crates/drawers can increase environmental humidity due to water evaporation from the respiratory tract and skin of the birds, and through moisture in excreta [32], however the stocking density of the crates/drawers in this study was unknown, as to avoid disturbing the animals, the crates and drawers were not opened during the observations. Although an effort was made to observe as many birds as possible, it was impossible to view each one, and as such, the total number of birds could not be counted, and the recorded number of crates/drawers containing panting birds may not have been a true representation. 

It is unsurprising that operator position was a significant predictor on the number of birds vigorously flapping in both processing plants. Loss of visual contact with other birds is an important cause of flapping at shackling [10]. Operators working at the position furthest from the water bath (position 6 in plant P1 and position 7 in plant P2) were placing birds on an empty shackle line, therefore there is no calming effect of neighbouring birds. In contrast, those working at position 1, closest to the water bath, were hanging birds on a shackle line which was already almost full of birds. It was observed that in both plants, the operator furthest from the water bath was responsible for ensuring that all the crates/drawers were empty of birds before they entered the washing area. The number of birds in each crates/drawers was not uniform, and therefore, if surplus birds were present in the crates/drawers when they reached the final position, these operators were required to shackle multiple numbers of birds. It was often observed that this resulted in an increase in rough handling. When investigating the interaction effect of position × visit in processing plant P1, it was decided to explore further flapping at positions 1, 3 and 6 to assess the effects of training at the beginning, middle and end of the shackle and to account for effects of line fill on flapping. Position 6 had the biggest decrease in percentage of birds flapping from pre to post training visits. This may be due to the higher baseline reading during pre-T, or perhaps the improved handling techniques of operators may elicit a greater effect in this position, due to the lack of the calming effect of other birds on the shackle line, previously described. 

It is important to note that the operators rotated their position on the shackle line throughout a shift, therefore the individual working at position 1 on day one of a visit may be working at a different position when assessed on a subsequent day. Due to clothing and PPE requirements, it was not possible to identify individual operators, therefore the presence of particularly “rough” or “good” practice by an individual may have influenced the results.

Training had a positive impact on the overall incidence of PSS, and the incidence of severe PSS in both processing plants. At the start of visit post-T the management team of plant P1 were eager to show the observers the new water-bath entry ramp that had been constructed as a result of the knowledge gained during the training course. This likely explains the reduction in PSS. The observers did note however, that at the beginning of visit 6mpost-T, the ramp had been modified after the previous visit, which resulted in an increase in PSS. Although levels were significantly lower than those of pre-training, this highlights the importance of continually monitoring welfare measures during the pre-slaughter and slaughter process and manipulating the process accordingly. 

Plant P2 did not install a new device at the entry to the water-bath, however management did report that they had increased PSS monitoring as a result of the training and adjusted the height of the water-bath in attempt to reduce PSS occurrence. 

It is encouraging that the training resulted in a significant increase in the applied current per-bird in the water-bath stunner, however, even post-training, both processing plants were not supplying sufficient current to effectively stun broilers [17]. It is generally agreed that increased stunning currents can lead to downgrading of the final product, including increased breast muscle haemorrhages and bone fractures [33,34,35]. This is not considered an animal welfare issue as birds are rendered unconscious immediately upon entry. However, associated product quality problems may result in stunning current being set too low, to reduce these downgrading conditions and thereby result in poor bird welfare [36]. 

The frequency used in plant P2 is higher than that recommended by Raj, O’Callaghan and Knowles [17], as this frequency was chosen in order to meet halal stunning requirements and is therefore unlikely to change as a result of the training. 

Training resulted in a marked improvement in the quality of neck cutting in plant P1. During pre-T, six out of the sample of 50 birds had both carotids intact post neck cut. It is likely that these birds would have regained consciousness prior to the scalding process [18]. However, it is possible that due to the low stunning currents used in plant P1, birds were electrically immobilised, rather than unconscious after exit from the water-bath [17]. All birds examined during the post training visits had at least one carotid severed, and a far greater proportion had both arteries severed, reducing the welfare risk of birds regaining consciousness during bleeding. At plant P1 the observers were informed by managerial staff that the automatic neck cutters, previously set to sever the necks had been readjusted following information given during the training. 

The variation in improvement between the processing plants may be attributed to several factors. Baseline measurements indicate that there was a higher standard of bird welfare prior to training in the UK processing plant when compared to that in Costa Rica. The importance attributed to animal welfare varies between countries: due to differences in traditions, religion, education, perception and level of economic development and legislation [37,38]. 

Legislative requirements regarding welfare at slaughter in Costa Rica and the UK differ. Slaughter facilities in the UK (England) must comply with The Welfare of Animals at the Time of Killing (England) Regulations 2015 [39] and Council Regulation (EC) No. 1099/2009 on the protection of animal at the time of killing [40]. These regulations outline numerous welfare requirements, including; minimum stunning currents, the requirement of the severing both carotid arteries at bleeding and the obligation to provide training to those handling live animals. The EU legislation also stipulates the requirement for specifically qualified “Animal Welfare Officers” in slaughterhouses. The Animal Welfare Officer is responsible for implementing animal welfare operating procedures and documenting action taken to improve animal welfare in the slaughterhouse. In contrast, the equivalent Costa Rican legislation [41] provides only stunning current recommendations, and stipulates that only one carotid artery needs to be severed at neck cutting. There is also no requirement for an Animal Welfare Officer. However, the Costa Rican legislation does include the requirement that operators handling live birds require training. Such legislative discrepancies may provide some explanation of the differences in the baseline welfare measurements in this study, especially regarding stun parameters and neck cutting. 

It is important to consider that in order to meet legislative requirements, both processing plants involved in this study had welfare training programs in place prior to the onset of the visits. Two members of the management team at processing plant P2 had previously attended a University of Bristol, two-day comprehensive poultry welfare training course, however none of the other attendees had experience of a similar course. 

It has been observed in Brazilian beef farms that training stockpeople regarding “good practice” is associated with both better attitudes and behaviours towards animals [42]. In this study, plant management, alongside operatives received welfare training. Although the managerial staff within a slaughter facility rarely handle animals, their attitudes have a significant influence on the welfare conditions within an abattoir [43]. Some of the welfare improvements in this study, for example, the reduction of vigorous wing flapping at shackling, are likely to be as a direct consequence of an operator behaviour change, others, such as increasing stun current in the water bath are likely to be implemented by managerial members of staff. 

A number of measures included in our study for example, stun parameters, PSS and neck vessels severed, which although showed improvement immediately after training (post-T) did not maintain such improvements six months later (6mpost-T). Paranhos da Costa, Huertas, Gallo and Dalla Costa [27] reported the results of a supermarket initiative in which beef farmers were trained in animal welfare. The training program resulted in a significant reduction in the proportion of downgraded carcasses due to bruising, however six months post training there was an increased percentage of downgraded carcasses. Turnover of staff, where trained staff may have left the processing plant after training and been replaced with untrained staff was not recorded in our study and this may have influenced the long-term changes in welfare outcomes.

It should be noted that in our study, flapping at shackling in processing plant P1 did continue to significantly decline between post-T and 6mpost-T, suggesting that certain positive behaviours of operatives may have become routine, or had been regularly reinforced by management.

### 4.2. Product Quality

In this study, the effects of training on product quality were somewhat more varied than that of the welfare outcome measures. Incidences of broken wings and red pygostyles appeared to decrease post training, while bruised legs and red wing tips increased. Producing high quality poultry meat at a commercial primary processing line requires a multi-factorial approach [6]. Although there is a well-documented link between bird welfare at slaughter and meat quality [8], carcass bruising can also be effected by other pre-slaughter factors, prior to arrival at the processing plant, such as catching and transportation [44]. Hamdy et al. [45] reported that approximately 90% of bruises found on broilers in American processing plants occur within the 13 h prior to slaughtering. A more recent Canadian study found that 5.7% of broilers per load, arriving at the slaughterhouse had wing damage [46]. Jacobs, Delezie, Duchateau, Goethals and Tuyttens [28] reported that the incidence of bruised wings tended to differ among different professional catching crews. Training catching crews in “better practice” can improve carcass quality [47] however this was beyond the scope of the current study.

Other factors, not necessarily associated with training, may have influenced results, for example bird factors such as age, sex and weight [48], loading conditions [28], environmental conditions [28,32], time of day of transportation [32], length of transport time [49] and crates/drawers stocking density [50] are all known to have effects on bruising and meat quality. These variables were not controlled by the methodology of this study.

It is unclear why bruising prevalence, especially regarding red wing tips and wing haemorrhage, differed in response to training between processing plant P1 and processing plant P2. Wing flapping and PSS are associated with wing damage [12,51]. Results from our study suggest that training improved the incidence of flapping and PSS in processing plant P1, yet the proportion of birds with economically significant red wing tips increased. Although both welfare and product quality assessments occurred on the same day, due to the logistical constrains of working in a high throughput commercial facility, different individual birds were included in the welfare and product quality observations. There is a potential that the physical presence of an observer during the welfare assessments affects the behaviour of processing plant personnel who “improve their performance” during the observation period, but revert back to normal practice when they are no longer being watched [52]. This “Hawthorne effect” (the alteration of behaviour by the subjects of a study due to their awareness of being observed) may have positively influenced the results of the welfare assessment, without affecting quality measurements. Further work is warranted to explore how welfare training, of the entire broiler production chain, may affect product quality.

### 4.3. Methodological Considerations

The primary processing plants we studied were, to a great extent, selected on their availability and willingness to participate in the study. The variation between the plants implies that this study might not give a complete picture of the effects of training across a wider selection of poultry slaughter facilities.

The recorded welfare and product quality measurements were selected based on a pilot study and on experience of the authors of slaughter in the UK and elsewhere. It was imperative that observations did not interfere with normal production and so some welfare assessment measures, such as effective stunning, could not be included in the protocol due to limited access to birds in the bleeding area in both plants.

## 5. Conclusions

Training in animal welfare does not take a fixed form—the type, depth and intensity of training depends very much on the needs of those to be trained, however it is recognised that training in the concepts of animal welfare within animal production builds the “capacities” of trainees [53]. In conclusion, our study supports the view that animal welfare training of stockpeople and managerial staff in commercial poultry primary processing plants has the potential to positively influence aspects of animal welfare and product quality. Legislation, retailer specifications and individual plant culture also play an important role and should be considered by those delivering welfare education in the slaughter industry.

## Figures and Tables

**Table 1:** Characteristics of the poultry plants involved in the study.

Plant	P1	P2
Processing speed (birds per hour)	10,500	10,400
Processing times	7:00 p.m.–10:00 a.m.	6:00 a.m.–4:00 p.m.
Weight of birds slaughtered (kg)	1.3–3	1.2–2.9
Breed of birds slaughtered	Ross/Cobb mix	Ross
Maximum bird transport time	4 h	3 h
Birds containment	Crates	Drawers
Neck cut method	Simmonds automatic neck cutter	Simmonds automatic neck cutter
Certified Halal	No	Yes

**Table 2:** The temperature (°C) and relative humidity (%) in the lairage as measured at the start of the welfare assessment.

		Temp °C/Relative Humidity%		
Processing Plant	Visit	Day 1	Day 2	Day 3
P1	pre-T	24.7/76.2	23.8/73	24.6/67.4
P1	post-T	25.2/56.5	23.6/58.9	24.7/61.9
P1	6mpost-T	32/94.2	23.2/89.6	25/72.8
P2	pre-T	25/66.5	23.3/64.9	24.3/74.9
P2	post-T	22.6/54.5	20.9/72.6	22.2/58
P2	6mpost-T	13.8/77.5	15.8/64.2	11.3/62.3

**Table 3:** Results of exact Kendall’s tau-b test of association between pre-stun shocks (PSS) and different visits.

Visit	Processing Plant P1	Processing Plant P2
pre-T–post-T	τb = −0.388, *p* < 0.005	τb = −0.009, *p* = 0.605
pre-T–6m-postT	τb = −0.172, *p* < 0.005	τb = −0.091, *p* < 0.005
post-T–6m-postT	τb = 0.24, *p* < 0.005	τb = −0.082, *p* < 0.005

**Table 4:** The percentage of birds in each visit to processing plant P1 with broken wings, bruised legs and within each external carcass quality class (0–2 or 0–3). Class 0 indicates no visible damage and class 1 indicates a low level of damage which will not result in downgrading while classes 2 and 3 indicate damage which will result in carcass downgrading [15].

**Visit**	**Broken Wings (n = 600)**	**% Birds with Red Pygostyles (n = 600)**			**% Birds with Shoulder Haemorrhage (n = 600)**				
	%	0	1	2	0	1	2	3	
pre-T	5.83	21.8	51.1	27	93	3.17	1.33	2.5	
post-T	3.17	43.5	42.8	13.67	94.83	3.33	1.5	0.33	
6mpost-T	3.17	34.8	48.6	16.5	90.83	4.5	2.5	2.17	
**Visit**	**Bruised Legs (n = 1200)**	**% Birds with Red Wing Tips (n = 600)**				**% Birds with Wing Haemorrhage (n = 600)**			
	%	0	1	2	3	0	1	2	3
pre-T	5.33	20.5	56.5	16.83	6.17	58.5	16.5	12.67	12.33
post-T	9.25	29.5	40.5	19	11	56.67	20.33	14.17	8.83
6mpost-T	17	15	56.17	19	9.8	53.83	21.17	18	7

**Table 5:** The percentage of birds in each visit to processing plant P2 with broken wings, bruised legs and within each external carcass quality class (0–2 or 0–3). Class 0 indicates no visible damage and class 1 indicates a low level of damage which will not result in downgrading while classes 2 and 3 indicate damage which will result in carcass downgrading [15].

**Visit**	**Broken Wings (n = 600)**	**% Birds with Red Pygostyles (n = 600)**			**% Birds with Shoulder Haemorrhage (n = 600)**				
	%	0	1	2	0	1		2	3
pre-T	8.5	21	39.83	39.17	85.83	10		3.17	1
post-T	4.33	25.33	50.17	22.83	87.83	9.33		2	0.83
6mpost-T	2.5	23	49.67	27.33	89.5	8.17		1.67	0.67
**Visit**	**Bruised Legs (n = 1200)**	**% Birds with Red Wing Tips (n = 600)**				**% Birds with Wing Haemorrhage (n = 600)**			
	%	0	1	2	3	0	1	2	3
pre-T	12.83	25.17	54	15.33	5.5	40.5	29.17	19.83	10.5
post-T	5.08	30.17	47.33	16.5	6	51.83	35.67	9.83	2.67
6mpost-T	16.67	39.17	52.67	7	1.17	58.5	26.17	11.5	3.83

**Table 6:** The test of association between the measurements and visits for the exact Chi-square test (Broken Wings and Bruised Legs) and exact Kendall’s tau-b test (remaining measures).

**Visit**	**Broken Wings**		**Red Pygostyles**		**Shoulder Haemorrhage**	
	P1	P2	P1	P2	P1	P2
pre-T–post-T	χ2(1) = 4.964, *p* = 0.036	χ2(1) = 8.673, *p* = 0.004	τb = −0.234, *p* < 0.0005	τb = −0.137, *p* < 0.0005	τb = −0.04, *p* = 0.161	τb = −0.031, *p* = 0.283
pre-T–6m-postT	χ2(1) = 4.964, *p* = 0.036	χ2(1) = 20.779, *p* ≤ 0.0005	τb = −0.159, *p* < 0.0005	τb = −0.93, *p* = 0.001	τb = 0.038, *p* = 0.181	τb = −0.056, *p* = 0.48
post-T–6m-postT	χ2(1) = 0, *p* = 1.0	χ2(1) = 3.056, *p* = 0.111	τb = 0.082, *p* = 0.003	τb = 0.046, *p* = 0.092	τb = 0.079, *p* = 0.006	τb = −0.026, *p* = 0.359
**Visit**	**Bruised Legs**		**Red Wing Tips**		**Wing Haemorrhage**	
	P1	P2	P1	P2	P1	P2
pre-T–post-T	χ2(1) = 13.616, *p* < 0.0005	χ2(1) = 44.186, *p* < 0.0005	τb = −0.147, *p* < 0.0005	τb = −0.006, *p* = 0.825	τb = −0.001, *p* = 0.978	τb = −0.166, *p* < 0.0005
pre-T–6m-postT	χ2(1) = 82.328, *p* < 0.0005	χ2(1) = 7.012, *p* = 0.01	τb = −0.19, *p* < 0.0005	τb = 0.058, *p* = 0.002	τb = 0.02, *p* = 0.471	τb = −0.192, *p* < 0.0005
post-T–6m-postT	χ2(1) = 31.605, *p* < 0.0005	χ2(1) = 83.060, *p* < 0.0005	τb = −0.155, *p* < 0.0005	τb = 0.079, *p* = 0.003	τb = 0.022, *p* = 0.417	τb = −0.041, *p* = 0.141
